# On the selection of the number of beamformers in beamforming-based binaural reproduction

**DOI:** 10.1186/s13636-022-00238-7

**Published:** 2022-03-30

**Authors:** Itay Ifergan, Boaz Rafaely

**Affiliations:** School of Electrical and Computer Engineering, Ben-Gurion University of the Negev, Be’er Sheva, Israel

**Keywords:** Binaural reproduction, Microphone arrays, Beamforming, Higher order Ambisonics, Directivity factor

## Abstract

In recent years, spatial audio reproduction has been widely researched with many studies focusing on headphone-based spatial reproduction. A popular format for spatial audio is higher order Ambisonics (HOA), where a spherical microphone array is typically used to obtain the HOA signals. When a spherical array is not available, beamforming-based binaural reproduction (BFBR) can be used, where signals are captured with arrays of a general configuration. While shown to be useful, no comprehensive studies of BFBR have been presented and so its limitations and other design aspects are not well understood. This paper takes an initial step towards developing a theory for BFBR and develops guidelines for selecting the number of beamformers. In particular, the *average directivity factor* of the microphone array is proposed as a measure for supporting this selection. The effect of head-related transfer function (HRTF) order truncation that occurs when using too many beamformer directions is presented and studied. In addition, the relation between HOA-based binaural reproduction and BFBR is discussed through analysis based on a spherical array. A simulation study is then presented, based on both a spherical and a planar array, demonstrating the proposed guidelines. A listening test verifies the perceptual attributes of the methods presented in this study. These results can be used for more informed beamformer design for BFBR.

## Introduction

Spatial audio has become a common feature in many application areas, such as virtual reality, hearing aids, teleconferencing, and entertainment. These applications use spatial audio to improve speech intelligibility or to spatially reproduce an acoustic scene for an immersive auditory experience.

A common format for spatial sound reproduction is higher order Ambisonics (HOA), which has lately been incorporated into the MPEG coding standard [1]. This approach aims to reproduce the sound pressure at the ears by employing spherical harmonics (SH) representations and using headphones [2] or loudspeaker arrays [3] for playback. The method became popular due to its convenient mathematical framework and its ability to capture HOA signals from a wide range of spherical microphone array configurations [4–7]. In addition, spatial audio recording and reproduction based on HOA has been well studied and its behavior and limitations are reasonably well understood [8–13]. HOA signals are also employed for binaural reproduction, where spherical microphone arrays [14, 15] are used to capture information about the sound field, which is then combined with head-related transfer function (HRTF) [16] representations in the SH domain. However, for spherical microphone arrays with a small number of microphones and a low SH order, the spatial resolution becomes poor and the quality of the spatial audio is degraded [13]. While many studies have been done on this subject [17–21], with the objective of improving the spectral coloration due to SH order truncation, leading to improved perception, the spatial properties of the signal have not been significantly improved. Furthermore, the method is not applicable to those arrays of general configurations which cannot provide HOA signals.

Alternative approaches which do not require spherical arrays have recently been developed. One such approach is the virtual artificial head (VAH) [22, 23], designed for anechoic environments but was recently studied for reverberant environment [24], which is based on applying beamforming to the microphone signals leading to binaural reproduction. Another similar approach, called beamforming-based binaural reproduction (BFBR), has been studied in [25, 26]. This approach was originally applied for spherical microphone arrays by using beamforming for plane-wave decomposition (PWD) at selected directions, followed by convolving the beamformer outputs with HRTFs in the same directions. This is similar to the virtual loudspeaker approach which is typically employed on the HOA signal [15], whereas for BFBR the application of beamforming does not necessarily incorporates, or leads to HOA signals, although this may depend on the beamforming design and the microphone array used. Other approaches, similar to BFBR, were also previously investigated in [27, 28] in which loudspeaker encoding techniques were applied on the Ambisonics signal, typically captured from a B-Format microphone array [29], instead of beamforming. Several studies employing BFBR then followed. The authors of [30] performed a psychoacoustic evaluation of this approach in a study of target detection in noise, as an alternative to binaural recordings from dummy heads. In [31], their research was extended to investigate the use of BFBR in the psychoacoustic evaluation of multichannel loudspeaker array configurations. The authors of [32] applied BFBR with maximum white noise gain beamformers, using a helmet-mounted microphone array for hearing protection while maintaining auditory situational awareness. Recently, the authors of [33] studied the error of binaural reproduction when increasing the number of beamformers, using BFBR with spherical arrays. Their results suggested that increasing the number of beamformers leads to a decrease in reproduction error and that this effect is saturated at some number of beamformers. However, their results are observatory only and limited to spherical arrays. Furthermore, no psychoacoustical evaluation has been presented to support this claim. Another relevant recent study on binaural reproduction with general array configurations was presented in [34], where the authors proposed a design based on the matching of the array output to binaural signals. However, the work only discussed synthetic sound fields with a finite number of incoherent sources and was not applied or studied with realistic sound fields, nor verified with a listening test. While these recent publications show encouraging results for BFBR, no comprehensive theoretical formulation or analysis of the approach has been presented so far, and nor has it been compared to the HOA-based binaural reproduction approach. Furthermore, it is not yet clear what are the limitations, or important parameters, affecting this approach.

This work makes an initial contribution towards the development of a theoretical design framework for BFBR. A set of guidelines for selecting the number of beamformers in BFBR is developed, employing a novel measure that leads to an informed selection of the number of beamformers. The consequences of selecting too many beamformers are also studied. First, the theoretical relation between BFBR and HOA-based reproduction is formulated for spherical arrays. Then, an appropriate choice for the number of beamformers is shown to be directly related to the directivity factor (DF) of the maximum directivity (MD) beamformer of spherical arrays. It is then proposed to generalize this measure for general arrays by quantifying the *average directivity factor* of the beam patterns. Next, the use of a larger number of beamformers is discussed and analyzed, showing that it can lead to order truncation of the HRTF and attenuation of high frequencies. Objective analysis using computer simulations was performed based on spherical and planar arrays, and a listening test was conducted to validate the results.

## Overview of BFBR

This section presents the mathematical background necessary for understanding BFBR. The first part of this section introduces a model of the sound pressure measured by general microphone arrays. Then, the BFBR approach is presented for the case of microphone domain beamforming.

Consider a microphone array positioned in a sound field with *S* far-field sources. The sound pressure at the *Q* microphones is measured at wave number *k*, where $k=\frac {\omega }{c}$, with *c* denoting the speed of sound propagation and with *ω* denoting the radial frequency. The sound pressure vector can be written as

1 $$ \mathbf{p}=\mathbf{V}\mathbf{s}+\mathbf{n}{,}  $$

with

2 $$ {\displaystyle \begin{array}{cc}\mathbf{p}=& {\left[\begin{array}{cccc}{p}_1(k)& {p}_2(k)& \cdots & {p}_Q(k)\end{array}\right]}^T\\ {}\mathbf{s}=& {\left[\begin{array}{cccc}s\left(k,{\varOmega}_1\right)& s\left(k,{\varOmega}_2\right)& \cdots & s\left(k,{\varOmega}_S\right)\end{array}\right]}^T\\ {}\mathbf{n}=& {\left[\begin{array}{cccc}{n}_1(k)& {n}_2(k)& \cdots & {n}_Q(k)\end{array}\right]}^T,\\ {}\end{array}} $$

and

3 $$ {\displaystyle \begin{array}{c}\mathbf{V}=\left[\begin{array}{cccc}\mathbf{v}\left(k,{\varOmega}_1\right)& \mathbf{v}\left(k,{\varOmega}_2\right)& \cdots & \mathbf{v}\left(k,{\varOmega}_S\right)\end{array}\right]\\ {}\mathbf{v}\left(k,\varOmega \right)={\left[\begin{array}{cccc}{v}_1\left(k,\varOmega \right)& {v}_2\left(k,\varOmega \right)& \cdots & {v}_Q\left(k,\varOmega \right)\end{array}\right]}^T,\\ {}\end{array}} $$

where *n*_*q*_(*k*) is the measurement noise of microphone *q* at wave number *k*. *s*(*k*,*Ω*) is the representation of all far-field source signals positioned at direction *Ω*, with *v*_*q*_(*k*,*Ω*) denoting the acoustic transfer function between *s*(*k*,*Ω*) and the pressure *p*_*q*_(*k*) measured by microphone *q*. The vector **v**(*k*,*Ω*) denotes the acoustic transfer function from source direction *Ω* to the microphone array. $\mathcal {S}\triangleq \{\Omega _{\xi }\}_{\xi =1}^{S}$ is the set of source directions of arrival with the *ξ*^*th*^ direction given by *Ω*_*ξ*_=(*θ*_*ξ*_,*ϕ*_*ξ*_), where *θ*_*ξ*_ denotes the elevation and *ϕ*_*ξ*_ denotes the azimuth direction. The source signals can represent, for example, any combination of direct sound sources and their reflections from room boundaries. Note that throughout this paper, the measurement noise is assumed to be negligible and is therefore omitted for simplicity.

BFBR aims to reproduce binaural signals at the ears from signals captured by a microphone array. This is achieved by employing the spatial information derived when applying beamforming, also denoted as spatial filtering, on microphone array signals [35]. The beamformer output for a steering direction *Ω*_*d*_ and wave number *k* is computed by

4 $$ y(k,\Omega_{d})=\mathbf{w}^{H}\mathbf{p}{,}  $$

with the beamformer weight vector denoted as

5 $$ \mathbf{w} = \left[ w_{1}(k,\Omega_{d}) w_{2}(k,\Omega_{d}) \cdots w_{Q}(k,\Omega_{d}) \right]^{T}{.}  $$

After beamforming is performed at a given set of *D* steering directions $\mathcal {D}\triangleq \{\Omega _{d}\}_{d=1}^{D}$, BFBR is applied for the left and right ears by multiplying each output with the corresponding HRTF, as follows [26]:

6 $$ \hat{p}_{l,r}(k) = \sum_{d=1}^{D}\alpha_{d} h_{l,r}(k,\Omega_{d}) y(k,\Omega_{d}){.}  $$

Here, *y*(*k*,*Ω*_*d*_) is the beamformer output at steering direction *Ω*_*d*_, as presented in (4); *h*_*l*,*r*_(*k*,*Ω*_*d*_) denotes the HRTF due to a plane wave arriving from direction *Ω*_*d*_ at the left or right ear [16]; and $\mathcal {A} \triangleq \{\alpha _{d}\}_{d=1}^{D}$ denotes the set of weights associated with the set of steering directions $\mathcal {D}$, to be discussed later.

The BFBR Eq. (6) provides an approximation for the pressure at the ears, denoted as $\hat {p}_{l,r}(k)$, with an accuracy which may depend on the sound field, the set of the steering directions used, the type of beamformers applied, and the characteristics of the microphone array. A comprehensive investigation into the complex, multi-featured behavior and performance of the BFBR approach is sorely needed; this paper takes an initial step towards this by studying the selection of the number of steering directions when capturing complex sound fields with several sources and room reflections. Because such complex sound fields are of interest in this work, sound field formulations based on general representations are employed. This is in contrast to a simpler sound field with only a few known source directions, for which tailored beamformers can be designed.

## BFBR for spherical microphone arrays

In Section 2, BFBR was introduced for general microphone arrays. In this section, spherical microphone arrays are discussed within the BFBR framework. Spherical microphone arrays are widely used and studied due to their ability to perform decomposition of the sound field into SH, leading directly to the HOA signal. This property is utilized here in order to derive a relation between HOA-based reproduction and BFBR for such arrays. First, BFBR is applied to spherical microphone arrays. Then, conditions for the equivalence of BFBR and HOA-based reproduction are presented.

For a spherical array, the acoustic transfer matrix can be represented in closed form [14, 36]:

7 $$ \mathbf{V} = \mathbf{Y}_{\boldsymbol{\mathcal{Q}}}\mathbf{B}\mathbf{Y}_{\boldsymbol{\mathcal{S}}}^{H}{,}  $$

with

8 $$ \begin{aligned} &\mathbf{B} = \text{diag} \left(\mathbf{b_{n}}\right)\\ &\mathbf{Y}_{\mathcal{Q}} = \left[\mathbf{y_{nm}}(\Omega_{1}) \mathbf{y_{nm}}(\Omega_{2}) \cdots \mathbf{y_{nm}}(\Omega_{Q}) \right]^{T}\\ &\mathbf{y_{nm}}(\Omega) = \left[Y_{0}^{0} \left(\Omega \right) Y_{1}^{-1} \left(\Omega \right) \cdots Y_{N_{p}}^{N_{p}} \left(\Omega \right)\right]^{T}{,} \end{aligned}  $$

where $Y_{n}^{m} \left (\Omega \right)$ denotes the order *n* and degree *m* SH basis function [14], $\mathbf {Y}_{\mathcal {Q}}$ is referred to as a SH matrix and is composed of a set of SH vectors *y*_*nm*_(*Ω*) with the set of microphone directions on the sphere, $\mathcal {Q}=\{\Omega _{q}\}_{q=1}^{Q}$, and with the maximum SH order *N*_*p*_ denoting the array order. $\mathbf {Y}_{\mathcal {S}}$ is similarly defined, where $\mathcal {S}$ is the set of source directions described in (3). *b*_*n*_ is a vector containing the radial functions [14, 37] which are arranged with respect to the SH order of *y*_*nm*_(*Ω*). Therefore, for a spherical array, **V** can be represented in terms of the order-limited linear transformation from the SH subspace of the source signal directions to the SH subspace of the microphone directions. The sound pressure model for a spherical microphone array is therefore given by substituting (7) into (1):

9 $$ \mathbf{p} = \mathbf{Y}_{\boldsymbol{\mathcal{Q}}}\mathbf{B}\mathbf{Y}^{H}_{\boldsymbol{\mathcal{S}}}\mathbf{s} = \mathbf{Y}_{\boldsymbol{\mathcal{Q}}}\mathbf{B}\mathbf{a_{nm}}{,}  $$

with

10 $$ \mathbf{a_{nm}} = \left[ a_{00}(k) a_{1(-1)}(k) \cdots a_{N_{p}N_{p}}(k)\right]^{T}  $$

representing the HOA signal vector, limited by the array’s SH order *N*_*p*_.

The beamformer output for a spherical microphone array is computed by substituting the sound pressure in Eq. (9) into the beamformer output Eq. (4):

11 $$ y(k,\Omega_{d})=\mathbf{w}^{H}\mathbf{Y}_{\boldsymbol{\mathcal{Q}}}\mathbf{B}\mathbf{a_{nm}}{.}  $$

Next, a beamformer is designed to extract the HOA signal *a*_*nm*_ (i.e., perform PWD [14]). The MD beamformer has been previously shown to perform PWD of the sound field surrounding an array [14, 26, 38]. The weights for this MD beamformer can be derived by maximization of the DF, or by using the minimum variance distortionless response (MVDR) beamformer under the assumption of an additive diffuse noise field [39]. For the beamformer output in Eq. (11), the following weights perform PWD:

12 $$ \mathbf{w} = [\mathbf{y_{nm}}(\Omega_{d})^{T} \cdot (\mathbf{Y}_{\boldsymbol{\mathcal{Q}}}\mathbf{B})^{\dagger}]^{H}{.}  $$

Substituting (12) into (11) leads to

13 $$ y(k,\Omega_{d})=\mathbf{y_{nm}}(\Omega_{d})^{T} \cdot \mathbf{a_{nm}} \equiv \bar{a}(k,\Omega_{d}){,}  $$

with $\bar {a}(k,\Omega _{d})$ denoting the order limited plane-wave amplitude density function (PWADF) at direction *Ω*_*d*_, which is limited to order *N*_*p*_ in the SH domain.

BFBR for the spherical microphone array is now applied by substituting Eq. (13) for the set of steering directions $\mathcal {D}$ into the BFBR Eq. (6):

14 $$ \hat{p}_{l,r}(k) = \sum_{d=1}^{D}\alpha_{d} h_{l,r}(k,\Omega_{d})\bar{a}(k,\Omega_{d}){.}  $$

By assuming that the SH representation of the HRTF is limited to order *N*_*h*_, and by applying the sampling conditions for aliasing-free integration, as developed in [40], to the set of steering directions $\mathcal {D}$, and the set of weights $\mathcal {A}$ in (14), the BFBR Eq. (14) can be reformulated as an integral:

15 $$ p_{l,r}(k)=\int_{\mathbb{S}^{2}}h_{l,r}(k,\Omega) \bar{a}(k,\Omega) \mathrm{d}\Omega{,}  $$

with $\mathbb {S}^{2}$ denoting the surface of the unit sphere. For the reformulation above to hold, the sampling scheme used for the beamformer steering directions must support aliasing-free sampling of order *N*_*D*_= max{*N*_*p*_,*N*_*h*_} or higher. By applying Parseval’s Theorem for SH, followed by the complex conjugate relation [14] to (15) the sound pressure at the ears can be computed using SH representation as [2]

16 $$ p_{l,r}(k)=\sum_{n=0}^{N}\sum_{m=-n}^{n} (-1)^{m} h_{nm}^{l,r}(k) a_{n(-m)}(k){,}  $$

with *N*= min{*N*_*p*_,*N*_*h*_}. Here, $h_{nm}^{l,r}(k)$ and the HOA signal *a*_*nm*_(*k*) are the spherical Fourier transform (SFT) coefficients of the HRTF and of the PWADF, respectively, employed in (15). This is exactly the sound pressure at the ears, as produced by using the HOA-based reproduction approach [2].

Although this equivalence has only been derived for spherical arrays, or arrays that can perform PWD, this result motivates the derivation of a measure that aids in the selection of the number of beamformers for BFBR with general arrays. Such a measure is suggested in the following section. Furthermore, a known drawback of HOA-based reproduction for HOA signals of low order is the reduced quality, due to the spatial and temporal smoothing that is a result of the truncation to the HRTF SH order. The latter may greatly reduce the reproduction quality [12, 13]. Section 5 will demonstrate how such smoothing is also likely to occur when employing BFBR with general arrays.

## Sampling conditions for BFBR with general arrays

Sampling conditions for spherical arrays that guarantee BFBR to be equivalent to HOA-based reproduction have been presented in the previous section. These are outlined here as a starting point for the subsequent discussion:

(i) PWD beamformers are used.
(ii) The steering directions and their associated set of weights $\mathcal {D}$ and $\mathcal {A}$, respectively, must define a sampling set on the sphere that is aliasing-free to order *N*_*D*_= max{*N*_*p*_,*N*_*h*_}, where *N*_*p*_ is the order of the spherical array and *N*_*h*_ is the order of the HRTF.
(iii) Then, BFBR is equivalent to HOA-based reproduction of order *N*= min{*N*_*p*_,*N*_*h*_}.

However, arrays of general configurations cannot typically perform PWD, and so for such arrays, these sampling conditions are no longer useful. Therefore, we would like to develop a similar set of mathematical conditions for BFBR with general arrays, as such conditions do not exist in the literature. The development of a mathematically complete set of sampling conditions to support BFBR design with general array configurations is challenging and is suggested for future studies. Instead, in this paper, the sampling conditions presented above for spherical arrays are reformulated, and this reformulation forms the basis for the proposal of design guidelines for BFBR with general arrays.

### Reformulation of the sampling conditions for BFBR with spherical arrays

The first step in the reformulation is a simplification with regard to SH orders. Since the order of the array is commonly lower than that of the HRTF, the latter is truncated to equal the order of the array. This reduces the number of steering directions used without changing the reproduced order, i.e., *N*= min{*N*_*p*_,*N*_*h*_}. Second, because the SH order is a parameter that is available for spherical arrays, but not for general arrays, we propose substituting the DF of the MD beamformer, which is used for PWD, with the order, as the former can be computed for any array. Now, the DF of the MD beamformer of a spherical array is given by [14]

17 $$ DF(k,\Omega_{d}) = (N_{p}+1)^{2}{,}  $$

where the DF is generally defined as [35]

18 $$ DF(k,\Omega_{d}) = 4\pi\frac{\left| \mathbf{w}^{H}\mathbf{v}(k,\Omega_{d}) \right|^{2}}{\int_{\mathbb{S}^{2}}\left| \mathbf{w}^{H}\mathbf{v}(k,\Omega) \right|^{2}\mathrm{d}\Omega}{,}  $$

with **v**(*k*,*Ω*_*d*_) as in (3), and *Ω*_*d*_ is the beamformer steering direction. Using (17) leads to the following, reformulated but equivalent, sampling conditions for spherical arrays:

(i) MD beamformers are used
(ii) The steering directions and their associated set of weights $\mathcal {D}$ and $\mathcal {A}$, respectively, each with a cardinality of *D*, must define a sampling set on the sphere that leads to aliasing-free integration, satisfying *D*≥*DF*

These conditions are equivalent to the ones presented previously, since the minimum number of samples for integration on the sphere, that is aliasing-free up to order *N*_*p*_ is (*N*_*p*_+1)^2^ [41], which is exactly the value of the DF in this case(17). Therefore, also under these conditions, equivalence between BFBR and HOA-based reproduction is obtained.

### Selecting the number of beamformers for general arrays

The reformulated sampling conditions in Section 4.1 form the basis for conditions applied to general arrays. Although they only apply to spherical arrays, they present a relation between the DF and the number of steering directions that lead to good quality reproduction (HOA-based binaural reproduction). Here, we make the following important step: we propose to use the same relations for general arrays. This does not guarantee reproduction in the form of HOA-based reproduction, but could lead to a good design.

However, when BFBR is applied to general arrays, the DF of individual beamformers may depend on the steering direction and on frequency. To overcome this complexity, the *average directivity factor* is proposed as an alternative measure to the DF in the reformulated sampling conditions above and is given by

19 $$ DF_{avg}(k) = \frac{1}{4\pi}\int_{\mathbb{S}^{2}}DF(k,\Omega)\mathrm{d}\Omega{.}  $$

This now leads to sampling conditions that are similar to those presented above for spherical arrays:

(i) MD beamformers are used
(ii) The steering directions and their associated set of weights $\mathcal {D}$ and $\mathcal {A}$, respectively, each with a cardinality of *D*, must define a sampling set on the sphere that leads to aliasing-free integration, satisfying *D*≥*DF*_*avg*_(*k*)

This choice makes sense for a number of reasons. First, when using a sampling scheme that is directly related to the DF, which is related to the main lobe width, we expect the set of beams to cover the entire directional space with the appropriate amount of overlap. However, because the DF may change with the look direction, the standard deviation of the DF can be introduced as a useful measure:

20 $$ DF_{STD}(k)=\sqrt{\frac{1}{4\pi}\int_{\mathbb{S}^{2}}\left[DF(k,\Omega)-DF_{avg}(k)\right]^{2} \mathrm{d}\Omega}{.}  $$

Furthermore, it has been shown in [42, 43] that the average directivity factor of the MD beamformer along all directions is given by the number of microphones in the array, denoted by *Q*. This provides a useful simplification — the number of beamformers can be chosen to be equal to or larger than the number of microphones in the array. Therefore, the following even more general relation can replace the sampling conditions for a general array:

(i) MD beamformers are used
(ii) The steering directions and their associated set of weights $\mathcal {D}$ and $\mathcal {A}$, respectively, each with a cardinality of *D*, must define a sampling set on the sphere that leads to aliasing-free integration, satisfying *D*≥*Q*

The sampling conditions above were formulated by assuming MD beamformers because of the mathematical relations available for these beamformers. This is not to say that these conditions will not hold when using other beam patterns. Other beam patterns can also be applied in practice, but their study is beyond the scope of this paper and is proposed for future work. Furthermore, the average directivity factor may change with frequency. This implies that different frequencies may require different beamformer distributions when using BFBR. Also, frequency-dependent beamformers may lead to timbral distortion, which may affect the performance of BFBR. These issues are also beyond the scope of this paper and are left for future research.

### BFBR with high order HRTFs

The sampling conditions introduced above for BFBR with MD beamformers for general microphone arrays were based on the simplification that a low order HRTF, i.e., *N*_*h*_=*N*_*p*_, was used. However, the truncation of the order of the HRTF may reduce the spatial qualities, degrading spatial perception [2, 13, 15, 44]. A number of approaches such as Mag-LS [18], spectral equalization [44], or other approaches for spectral coloration correction [12, 19] may be used to improve perception with low order HRTFs. Furthermore, a study has recently compared the above methods [20]. The use of all these methods for BFBR is interesting, but may be beyond the scope of this paper. Here, however, we propose to use the high order HRTF directly without increasing the number of beamformers. The rationale behind this is that although violating the sampling condition (following [40]), the effect of the high orders on aliasing may be small due to the natural HRTF order roll-off, in particular at the lower frequencies where equivalence to HOA-based reproduction may still approximately hold [2, 40], and the overall effect on perception may be positive [2, 13, 44]. This was verified in an informal listening test.

This alternative is based on two conditions:

(i) The full high order HRTF is employed.
(ii) The reformulated sampling conditions in Section 4.1 are used where the number of beamformers *D* is chosen to be as close to the lower bound, *DF*_*avg*_(*k*), as possible.

In summary, the use of full-order HRTFs with the minimal number of beamformers aims to strike a balance between keeping high order spatial information and reducing spatial errors due to spatial aliasing. While only partially justified in theory, this approach is shown to be useful, as will become evident from the investigations reported in the following sections.

## The consequences of oversampling with high order HRTFs

In this section, the consequences of oversampling in BFBR are discussed. In the context of this work, oversampling is relative to the conditions in Section 4.3, in conjunction with the reformulated sampling conditions in Section 4.2.

### Oversampling leads to order truncation

Assume a beamformer is applied to a general array with an output given by *y*(*k*,*Ω*), as in (6). It is also assumed that the SH representation of *y*(*k*,*Ω*) is of limited order denoted by *N*_*y*_ (see formulation in the next subsection). The HRTF, *h*_*l*,*r*_(*k*,*Ω*), is also assumed to be limited to some high order *N*_*h*_, as discussed in Section 4.3. Similar to the case of spherical arrays, a dense beamformer distribution in (6) is assumed such that *D* is very large. The latter means that the beamformers’ steering directions and their appropriate weights represent a sampling scheme which is aliasing-free up to SH order *N*_*D*_≥ max{*N*_*y*_,*N*_*h*_}. Under these assumptions, (6) can be replaced by an integral over the sphere, and after applying Parseval’s relation, (6) can be rewritten as

21 $$ \hat{p}_{l,r}(k) = \sum_{n=0}^{N_{e}}\sum_{m=-n}^{n} (-1)^{m} h_{nm}^{l,r}(k) y_{n(-m)}(k){,}  $$

where *N*_*e*_= min{*N*_*y*_,*N*_*h*_} and *y*_*nm*_(*k*) are the SFT coefficients of the beamformer output function *y*(*k*,*Ω*).

The similarity between (21) and (16) is clear, where *y*_*nm*_(*k*) is replaced by *a*_*nm*_(*k*) in Eq. (21). Now, recall that (16) leads to order truncation, as noted above. It is therefore expected that *y*_*nm*_(*k*), if limited to a low order, *N*_*y*_, will lead to a similar effect, i.e., order truncation of the HRTF to order *N*_*y*_. This is further analyzed in the next subsection.

### Analysis of the order of *y*_*nm*_(*k*)

The function *y*(*k*,*Ω*) represents the way in which the beamformer output varies with look direction and therefore may depend on the design of the beamformers. With the aim of gaining insight into the behavior of *y*_*nm*_(*k*), the beamformers are assumed to belong to a common family of designs, including the MVDR (i.e., Capon), and MD, as detailed in Section 3. A general formulation for this beamformer family is given by [35]

22 $$ \mathbf{w}(k,\Omega_{d}) = c(k,\Omega_{d}) \cdot \left[\mathbf{R}(k)\right]^{-1} \cdot \mathbf{v}(k,\Omega_{d}){,}  $$

where *c*(*k*,*Ω*_*d*_) is a normalization coefficient, and **R**(*k*) is a noise covariance matrix. For the MVDR beamformer, **R**(*k*) represents the covariance of the noise signal, while for the MD beamformer, it is assumed that the noise signal is spatially white, or diffuse. Finally, for the Bartlett beamformer, **R**(*k*) is the identity matrix.

Substituting (22) into (4) and equating the normalization coefficient *c*(*k*,*Ω*_*d*_) to be 1 for simplicity lead to

23 $$ \begin{aligned} y(k,\Omega) &= \left[\mathbf{v}(k,\Omega_{d})\right]^{H}\left[\mathbf{R}(k)\right]^{-1}\mathbf{p}(k) \\ &= \sum_{q=1}^{Q} r_{q}(k) \cdot \left[ v_{q}(k,\Omega) \right]^{*}{,} \end{aligned}  $$

where *r*_*q*_(*k*) are the elements of the product of [**R**(*k*)]^−1^ and **p**(*k*).

It is clear from (23) that the beamformer output in this case is a linear combination of the acoustic transfer functions of the array microphones, *v*_*q*_(*k*,*Ω*). It is therefore expected that *y*_*nm*_(*k*) will inherit its SH order behavior from *v*_*q*_(*k*,*Ω*). As discussed in [37], for example, the SH behavior of the acoustic transfer function of a free-field microphone positioned at a distance *r*_*a*_ from the origin is dominated by radial functions (spherical Bessel and Hankel functions). This behavior typically exhibits a roll-off along the order *n*, with a cut-off which is approximately at *N*_*p*_=*kr*_*a*_. However, the latter may depend on whether the microphone is in free-field or placed around a rigid body, for example. On the other hand, [**R**(*k*)]^−1^ may lead to enhancement of higher orders at the expense of robustness [45], which may lead to noise-sensitive beamformers. Moreover, note that the rank of the matrix **R**(*k*) is limited by the number of microphones *Q*. This further limits the possible order of *y*_*nm*_(*k*).

This analysis leads to the conclusion that the order *N*_*y*_ of *y*_*nm*_(*k*) is therefore expected to be bounded, with the bound affected by the order of the array, *N*_*p*_, the type of beamformer, and the number of microphones.

### Computation of the order of *y*_*nm*_(*k*)

While the order of *y*_*nm*_(*k*) is expected to be limited, as argued in the previous subsection, it may be useful to compute it numerically for specific arrays. A numerical approach for the approximation of *N*_*y*_ is formulated next.

First, *y*_*nm*_(*k*) is computed from the beamformer output function *y*(*k*,*Ω*). After *y*_*nm*_(*k*) is obtained, an estimate of *N*_*y*_ is computed as follows:

24 $$ \begin{aligned} N_{y}(k) = \arg\min_{N} &\left\{\sum_{n=0}^{N}\sum_{m=-n}^{n}\left| y_{nm}(k) \right|^{2} > \right.\\ &\quad\left.\eta \cdot \sum_{n=0}^{\infty}\sum_{m=-n}^{n}\left| y_{nm}(k) \right|^{2} \right\}{,} \end{aligned}  $$

where *η* is chosen to be of a value close to 1, for which most of the power of the beamformer output is contained up to order *N*_*y*_(*k*). *N*_*y*_(*k*) can then be used to assess the truncation order of the HRTF when using BFBR.

## Measures of frequency-dependent performance

Truncation of the HRTF, as discussed above, may lead to attenuation at high frequencies. It is therefore desirable to measure the frequency-dependent power of the sound pressure at the ears when applying BFBR, in order to quantify such attenuation. Furthermore, it may also be instructive to compare the accuracy of BFBR relative to a HOA-based reproduction reference. Therefore, the relative mean square error (rMSE) for the BFBR signals is also presented.

To derive the first measure, Eq. (4) is substituted into the BFBR Eq. (6), and its expected power is computed, leading to

25 $$ \begin{aligned} E_{\hat{p}_{l,r}}(k) &= \mathbb{E}\left[\left| \hat{p}_{l,r}(k) \right|^{2}\right] = \mathbb{E}\left[\left| \mathbf{w_{h}}^{H} \mathbf{p} \right|^{2}\right] \\ &=\mathbf{w_{h}}^{H}\mathbf{R_{p}}\mathbf{w_{h}}{,} \end{aligned}  $$

with *R*_*p*_ denoting the covariance matrix of **p**, and

26 $$ \mathbf{w_{h}} = \sum_{d=1}^{D}\left[ \alpha_{d} h_{l,r}(k,\Omega_{d}) \right]^{*} \mathbf{w}{.}  $$

We recall that **w** is dependent on the steering direction *Ω*_*d*_ as in (5), and both **w** and **p** are frequency dependent.

This measure can be compared to the case of a high order spherical array as a reference, which is computed by replacing $\hat {p}_{l,r}(k)$ in (25) with the sound pressure computed using HOA-based reproduction, (16). For simplicity, it is assumed that the sound field is diffuse, in which case the expected power is formulated as a function of the HRTF coefficients:

27 $$ E_{p_{l,r}}(k) = \left\lVert\mathbf{h_{nm}}\right\rVert_{2}^{2}{,}  $$

with

28 $$ \mathbf{h_{nm}} = \left[h_{00}^{l,r}(k) h_{1(-1)}^{l,r}(k) \cdots h_{N_{h}N_{h}}^{l,r}(k) \right]^{T}  $$

representing the HRTF SFT coefficients vector, limited by the HRTF’s SH order *N*_*h*_. This simplified result is obtained since a diffuse sound field forms a white noise signal in the SH domain [14].

The rMSE for the BFBR signals, relative to a HOA-based reproduction signal (16), is simply given by

29 $$ rMSE(\hat{p}_{l,r}(k)) = \frac{\mathbb{E}\left[\left| \hat{p}_{l,r}(k) - p_{l,r}(k) \right|^{2}\right]}{\mathbb{E}\left[\left| p_{l,r}(k) \right|^{2}\right]}{.}  $$

When computing the rMSE for a diffuse sound field, the denominator of (29) can be replaced by Eq. (27), where *N*_*h*_ is set to the SH order *N* of the HOA-based reproduction signal. If the sound pressure **p** can be represented in the SH domain, such as in the case of rigid spherical arrays or free field microphone arrays [14], the numerator of Eq. (29) could also be represented in closed form.

Comparing (25) for the case of a diffuse sound field with different values of *D* to the expected power reference (27) can be useful for understanding the effect of the choice of the number of beamformers in BFBR on the frequency-dependent magnitude of the binaural signal; here, (29) can be further applied to estimate the signal’s accuracy.

## Simulation study with spherical and planar arrays

In this section, a simulation study is presented to illustrate the theory and insights developed in Sections 4 and 5. In particular, spherical and planar arrays are simulated using MATLAB version R2019a, with the planar array serving as an example of a non-spherical array configuration. The selection of the number of beamformers is analyzed, together with other factors, and demonstrates the theoretical results. The listening test in the following section complements the simulation study.

### Setup

Two microphone arrays were simulated as follows. A spherical microphone array, consisting of *Q*=12 uniformly distributed microphones on the surface of a rigid sphere with a radius of 2.12 cm, based on a t-design of order 2 [46] was used. The array can perform aliasing-free PWD up to 5150 Hz. A planar array, which was a uniform array in free field consisting of *Q*=9 microphones on a 3×3 grid with a distance of 1.5 cm between the microphones, was used. The array, encapsulated within a sphere with radius 2.12 cm, was positioned on the *yz* plane. This microphone configuration allows for an aliasing-free processing bandwidth of about 11,500 Hz, with the sound pressure within the array region accurately represented using a SH expansion of order *N*_*p*_=5 in this bandwidth, following the relation *N*_*p*_=*kr*_*a*_, as presented in Section 5.2. Both arrays were centered at the origin and were assumed to include negligible sensor noise. HRTFs from the Cologne HRTF compilation of the Neumann KU-100 [47] of order *N*_*h*_=32 were used in Section 7.4 for the computation of the expected pressure power, presented in Section 6. The analysis in this section was performed over the operating frequency range of the planar array for frequencies between 375 and 11,250 Hz.

### Methodology

For the spherical and planar arrays, the sound pressure at each microphone was computed using the SH expansion for the sound pressure on a rigid sphere and in free-field, respectively [14], with *N*_*p*_=5, from which the steering vectors described in (3) were derived. Then, the DF was computed using (18), leading to the computation of its statistics, in accordance with (19) and (20). The MD beamformer for the spherical array was computed using (12), by assuming *N*_*p*_=2, and by adding the minimal diagonal loading necessary for numerical matrix inversion. The MD beamformer for the planar array was computed in practice using the MVDR design framework [39], by assuming a diffuse sound field with unit variance [14], and by further following subsection 5.2, i.e., choosing *c*(*k*,*Ω*_*d*_) in (22) to be 1.

### Analysis of DF statistics

In order to illustrate the theory presented in Section 4, the spatial statistics of the DF are presented for the planar and spherical arrays. The average directivity factor (19) and its standard deviation (20) were computed by employing numerical integration on the sphere using a Gaussian sampling scheme [14] of order 60.

**Fig. 1:**
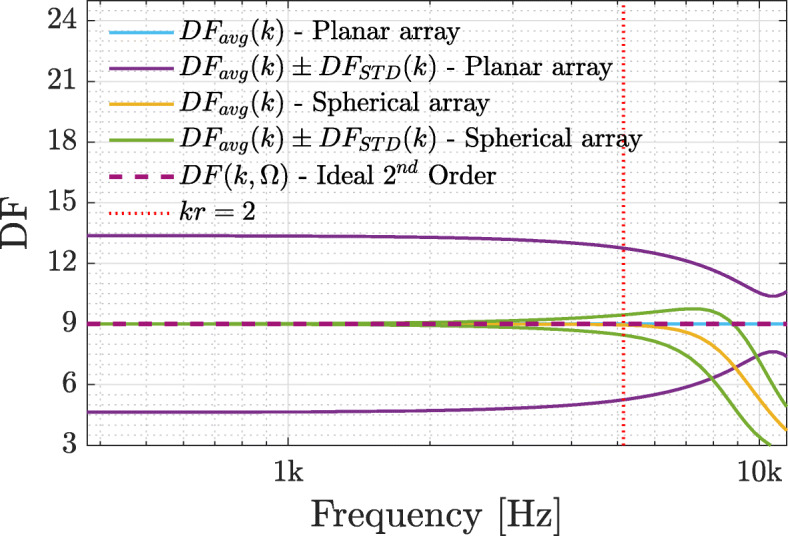
The average directivity factor (19) and its standard deviation (20) for the planar and spherical arrays, as described in Section 7.1

Figure 1 shows the average directivity factor and its standard deviation for the two arrays. The figure confirms that for the planar array the average directivity factor is equal to the number of microphones, *Q*=9, over the entire frequency range, as stated in [42]. The average directivity factor for the spherical array is also 9, as stated in (17), for a limited range; this is because of the significant spatial aliasing above this frequency range when applied to full order sound pressure. Above this frequency range, this beamforming design results in significant degradation to its directivity. Note that by computing the MD beamformers for the spherical array, with the same approach used for the planar array, which is not presented in Fig. 1, the average DF was indeed equal to the number of microphones, i.e., 12. This may imply that a beamformer design, similar to that of the planar array, may improve and extend the operating frequency range of the array. However, this is not further researched in this paper and is suggested for future study. The standard deviation for the planar array changes from ± 4.5 to ± 1.5 throughout the frequency range, while for the spherical array the standard deviation is zero, as expected from its constant DF (17) up until around the aliasing frequency of the array. The relatively large standard deviation of the planar array stems from its geometry, where the DF is higher for steering directions in the *yz* plane, the array end-fire direction, which is the plane of the array microphones. The reduced variability for the planar array at high frequencies is expected, since the distance between microphones approaches half a wavelength [35]. These computed values for the average directivity factor formed the basis for selecting the number of beamformers in the listening test, as suggested in Section 4.3, in conjunction with the sampling conditions in Section 4.2.

While the average directivity factor is used in this work for the selection of the number of beamformers, as described in Section 4, the standard deviation of the DF may also affect the selection process. However, further investigation of this is out of the scope of this paper and is left for future study.

### The effective order of *y*_*nm*_(*k*)

In the previous subsection, both arrays were shown to have an average directivity factor of 9. Following the discussion in Section 5, this means that using a much higher number of beamformers may lead to order truncation of the HRTFs, due to the order limitation of *y*_*nm*_(*k*). In this section, the effective order of *y*_*nm*_(*k*) is investigated in order to quantify and gain insight into the effect of order-truncation.

Recall that Eq. (21) in Section 5 presented *y*_*nm*_(*k*) as a modified plane-wave density function, taking the role of *a*_*nm*_(*k*) in (16). As discussed in Section 5.2, the behavior of *y*_*nm*_(*k*) may depend on the beamformer type. Therefore, in this study, MD vs. Bartlett beamformers are evaluated. The effective order of *y*_*nm*_(*k*) is computed in practice using (24), as described in Section 5.3.

**Fig. 2:**
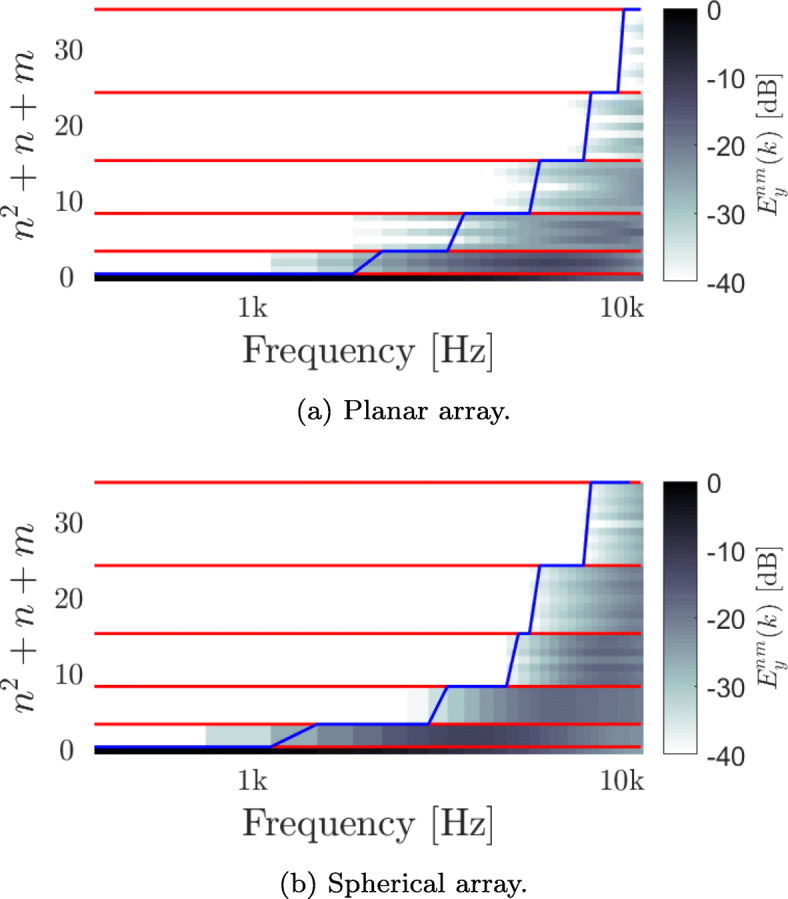
$E_{y}^{nm}(k)$, the expected power distribution of *y*_*nm*_(*k*) for the planar and spherical arrays with a diffuse sound field for a Bartlett beamforming design. The red lines, located at (*n*+1)^2^, differentiate between different orders. The blue line indicates the order *N*_*y*_(*k*), presented as (*N*_*y*_(*k*)+1)^2^, and computed as in (24) with *η*=0.99

**Fig. 3:**
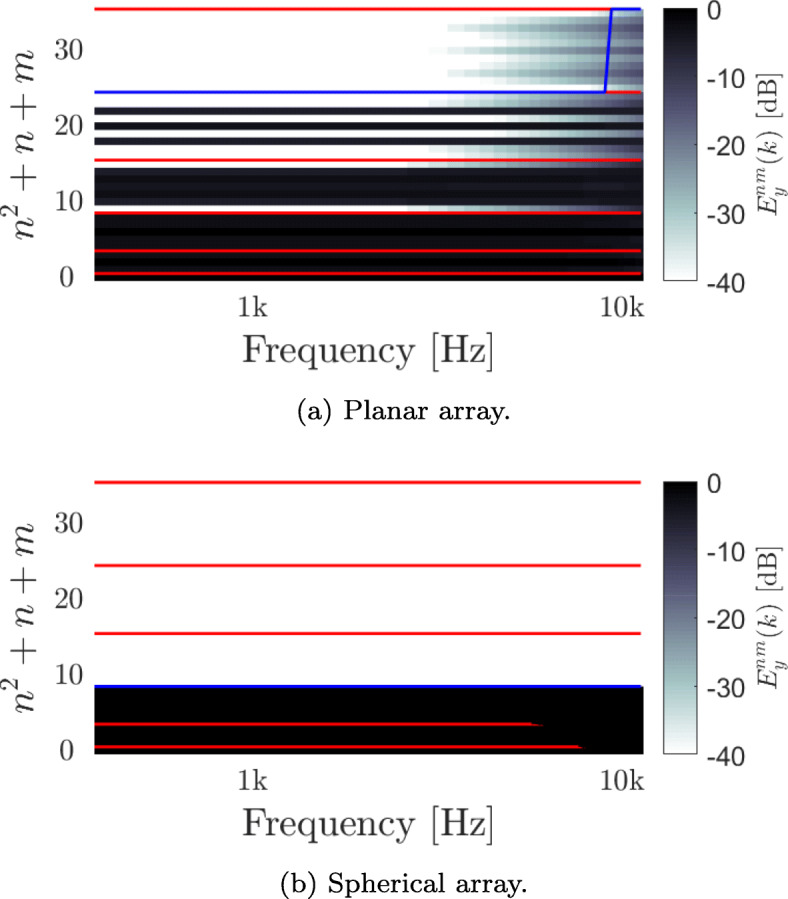
$E_{y}^{nm}(k)$, the expected power distribution of *y*_*nm*_(*k*) for the planar and spherical arrays with a diffuse sound field for a MD beamforming design. The red lines, located at (*n*+1)^2^, differentiate between different orders. The blue line indicates the order *N*_*y*_(*k*), presented as (*N*_*y*_(*k*)+1)^2^, and computed as in (24) with *η*=0.99

The effective order of *y*_*nm*_(*k*) was computed for the planar array described in Section 7.1, under the assumption of a diffuse sound field for the two beamformers, and is presented in Figs. 2 and 3. The red lines, located at (*n*+1)^2^, differentiate between values of (*n*,*m*) having the same order *n*. The blue line demonstrates the effective order, *N*_*y*_(*k*), at each frequency, presented as (*N*_*y*_(*k*)+1)^2^. *N*_*y*_(*k*) was computed using (24) with *η* chosen to be 0.99. Figures 2a and 3a show the results for the planar array, while Figs. 2b and 3b show the results for the spherical array.

Figure 2a shows the expected power, $E_{y}^{nm}(k)$, for the Bartlett beamformer, with its effective order, *N*_*y*_(*k*), growing with frequency up to *N*_*y*_(*k*)=5, as expected from the SH expansion of microphones in free-field. The very low order up to around 2000 Hz is a result of the beamformer’s robustness to sensor noise and the behavior of the SFT coefficients of the microphones’ acoustic transfer functions [45].

The spherical array, as presented in Section 7.1, is also presented in Fig. 2b for contrast with the planar array in Fig. 2a. Here, also, Bartlett beamformers were used. The behavior of the spherical array is similar to that of the planar array, as expected. Here, however, the first order is attained at a lower frequency. This is because the microphones of the spherical array have the same radius, which lead to a faster increase of attainable HOA order with frequency.

Figure 3a shows $E_{y}^{nm}(k)$ for the MD beamformer. In this case, *N*_*y*_(*k*) was computed to be 4 up to around 9000 Hz, followed by *N*_*y*_(*k*)=5 at higher frequencies. This behavior at the higher orders compared to the Bartlett beamformer can be explained by the dependency of the beamformer weights on the inverse of the covariance matrix **R**(*k*). This amplification of high orders at low frequencies leads to a high sensitivity to sensor noise, which is assumed to be negligible in this paper. Furthermore, note that, as discussed in Section 5.2, the maximal rank of the matrix **R**(*k*) is 9 in this case, therefore limiting the number of independent elements in *y*_*nm*_(*k*) to the same number. This, in turn, may impact perception. Similarly, a MD beamformer design with the spherical array is presented in Fig. 3b. As expected, the beamformer output is limited to order 2, which results from its beamforming design approach.

Since both beamformer designs were found to be order limited, it is expected that the use of too many beamformers in BFBR will lead to attenuation at higher frequencies due to the truncation of the HRTF order, as described in Section 5.1.

### Spectral analysis of BFBR with different numbers of beamformers

Having illustrated the order limitation of *y*_*nm*_(*k*), and having evaluated the average directivity factor to be 9, it is expected from the theory developed in this paper that HRTF order truncation will occur when selecting a number of beamformers that is significantly higher than 9. This is illustrated in this section.

**Fig. 4:**
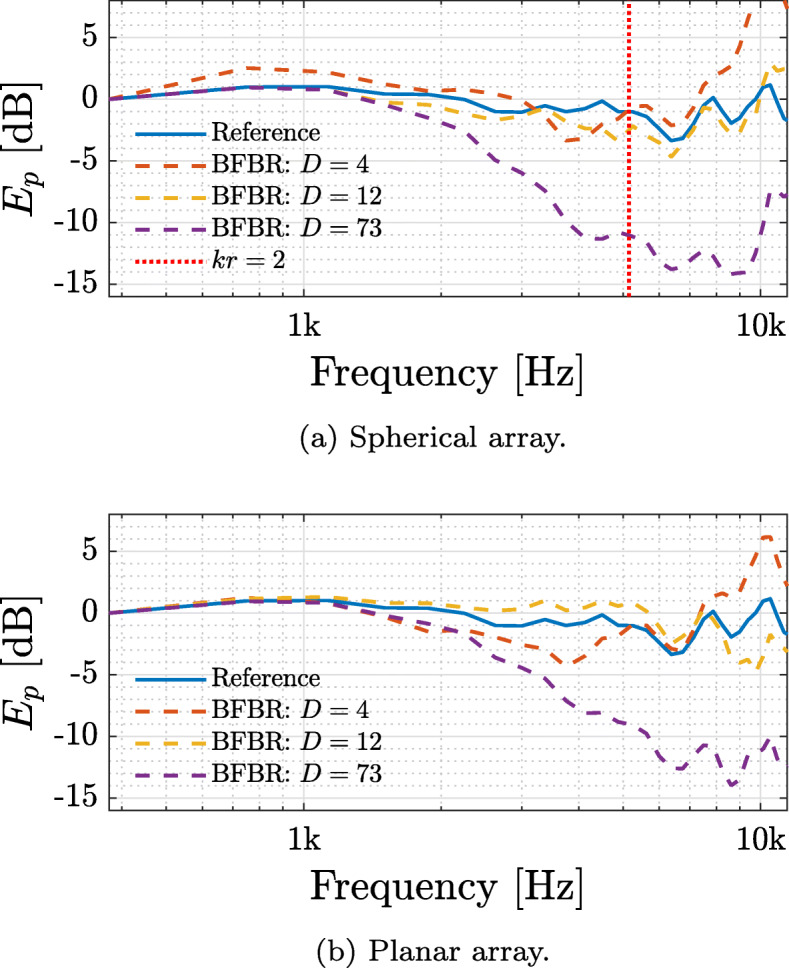
Expected power of the sound pressure at the left ear for a diffuse sound field, computed using (25), for BFBR with varying beamformer distributions, for the spherical and planar arrays. These are compared to a HOA-based reproduction reference of order 32, computed using (27). The signals were further normalized by their value at frequency 375 Hz

Three sets of beamformers were selected, i.e., 4, representing too few beamformers; 12, representing a number which is approximately the same as the average directivity factor; and 73, which is much higher. The beamforming directions were distributed uniformly, or nearly uniformly, according to appropriate t-designs, facilitating accurate integration on the sphere up to SH orders of *N*_*D*_=1, 2, and 5, respectively [46]. For the spherical and planar arrays, the expected power of the sound pressure at the ears was computed using (25) and was normalized with the value at frequency 375 Hz. The expected power for HOA-based reproduction signals of order 32 served as a reference and was computed using (27). Both were computed for the left ear and for both arrays and are presented in Fig. 4. The figure shows that for both arrays, increasing the number of beamformers beyond the average directivity factor (*D*=73 in the figure) leads to significant attenuation of high frequencies, as expected. Note that this roll-off at high frequencies is significant even though the condition *N*_*D*_≥ max{*N*_*y*_,*N*_*h*_}, discussed in Section 5.1, did not hold. Furthermore, using a number of beamformers which does not satisfy the conditions in Section 4, i.e., *D*=4 (smaller than 9), is shown to lead to significant amplification at high frequencies. Interestingly, BFBR with 12 beamformers is the most similar to the HOA-based reproduction reference. This implies that using a number of beamformers that is similar to the average DF, but not much larger, may lead to correct timbre in the reproduced signal. This latter attribute will be further demonstrated in the listening test in Section 8.

**Fig. 5:**
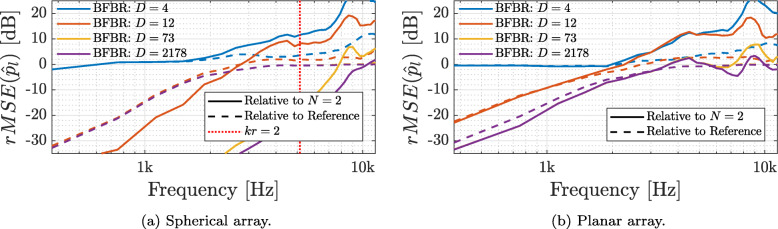
Relative MSE of the sound pressure at the left ear for a diffuse sound field, computed using (29), for BFBR with varying beamformer distributions, for the spherical and planar arrays relative to a HOA-based reproduction reference and to a 2nd order Ambisonics-based reproduction

To complement the analysis above, the *rMSE* of the BFBR signals was also computed, by applying Eq. (29) on both arrays, relative to the HOA-based reproduction reference presented previously in this section, and to 2nd order Ambisonics-based reproduction, i.e., the order of the spherical array, and is presented in Fig. 5. Here, BFBR with the same number of beamformers as presented above is used, with the addition of a BFBR signal with *D*=2178 beamformers, distributed using a Gaussian sampling scheme [14] of order 32 — the order of the HRTF, which leads to (21).

The figure shows that for both arrays the lower frequencies have a low rMSE when the sampling conditions discussed in Section 4 hold. This is expected, as was also discussed in Section 4 and in [40]. Interestingly, the error of the planar array at low frequencies is also relatively low, even though the array has front-back ambiguity. This may be related to the isotropic properties of the diffuse noise, and so the error may increase in other sound fields. The error relative to the HOA-based reproduction reference reaches saturation at higher frequencies, at around 0 dB for both arrays, while the error relative to the 2nd order Ambisonics-based reproduction becomes much higher at high frequencies. This is expected due to the missing higher orders in the 2nd order Ambisonics-based reproduction signal. These results, along with the previous results in this section, validate the choice of the number of beamformers in BFBR, as suggested in Section 4.

## Listening test

Two listening tests were performed to validate the theory derived in Sections 4 and 5 and to further evaluate spatial perception with BFBR. The tests were based on Recommendation ITU-R BS.1534-1 (MUSHRA, MUltiple Stimuli with Hidden Reference and Anchor) [48] and aimed to investigate the effects of the number of beamformers in BFBR on spatial and timbre perception. Due to the current circumstances of COVID-19, the listening test was performed virtually, with each participant performing the test at the place of their choice and with their own equipment.

### Setup

The simulated acoustic environment in this test was composed of a rectangular room of dimensions 9.8×15.5×7.5 m with a wall reflection coefficient of 0.8 and a reverberation time *T*_60_=0.75 s, resulting in a critical distance of *r*_*d*_=2.2 m. The sound field in the room was simulated using the image method [49], where a sampling rate of *f*_*s*_=48 kHz was employed.

A planar array was simulated in this room, along with a spherical array of order 2, as described in Section 7.1. The spherical array’s origin of coordinates was located 1 m from the wall, at a height of 1.5 m from the floor, at position (1,7.75,1.5) m, while the planar array was positioned on the wall at a height of 1.5 m from the floor, with the array center at (0,7.75,1.5) m. The array was positioned on the wall in order to avoid the ambiguity inherent in the directivity of this array. A single source was located at a distance of *d*=3*r*_*d*_ from each array, at an azimuth and elevation of $(\theta,\phi) = (\frac {\pi }{2},\frac {\pi }{4})$ relative to the array, i.e., at the same height and at 45^∘^ horizontally. Two types of source signals were used. The first signal was speech, taken from the McGill TSP speech database [50], and the second was composed of repeating pulses of noise, each pulse with a duration of 0.2 s, spaced by 0.33 s of silence.

**Fig. 6:**
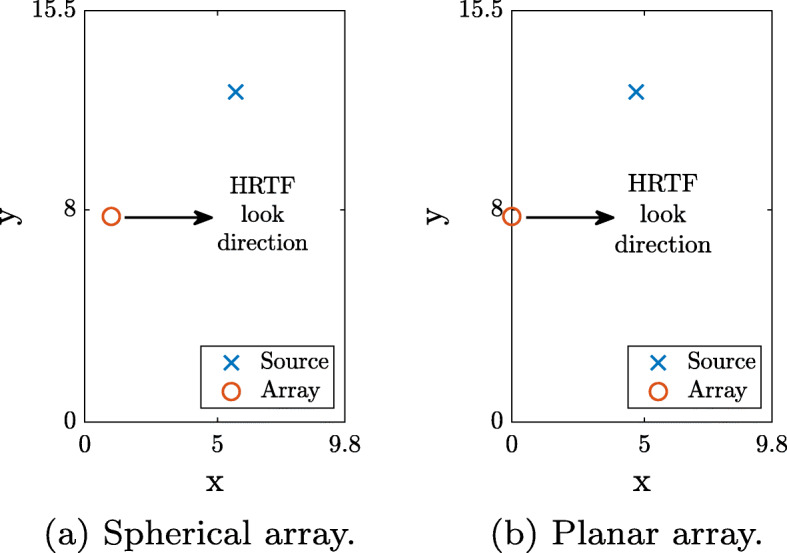
Room description for the spherical and planar array scenarios in the listening test. The location of the source is marked by “x,” the location of the array by “o,” and the HRTF look direction is marked using an arrow

The HRTF used in the tests was the same as that presented in Section 7.1, which was further equalized with a headphone compensation filter [47], selected according to the specific headphones of each user. The signals in the evaluation were played back directly from MATLAB using a set of three different headphones available to the participants, namely, AKG-K701/K702 and Beyerdynamic-DT990PRO. The source and array locations and the HRTF look direction are shown in Fig. 6.

### Methodology

The signals for the listening test were generated as follows. Room impulse responses (RIR) were computed for each microphone by first computing the HOA signal’s impulse response for each array’s origin of coordinates, which was then used to compute the sound pressure of the microphones using plane-wave composition representation [14], assuming free-field for the planar array. For the spherical array, the pressure was computed as in (9) with the HOA signal order limited to 2 to avoid the effect of aliasing. This simplification was introduced to ensure that the comparison between the arrays is focused on the beamforming-based reproduction aspect, rather than other array design aspects. The spherical array is compared to the planar array, as discussed in Section 7.1. Then, MD beamformers were applied to the microphone signals (the RIRs), as presented in Section 7.2, with the beamformers steered at various directions, as specified below. Finally, the beamformer outputs were filtered by the appropriate HRTFs, as described in (6), generating binaural room impulse responses (BRIRs). These were then convolved with anechoic source signals to produce the binaural signals. The BRIRs were computed at a single head rotation, i.e., the head is directed at $(\frac {\pi }{2},0)$. HOA-based BRIRs were produced by incorporating the RIR of the source signal as a HOA signal, previously computed for the microphone arrays, into (16), using the same HRTF, later to be convolved with the same anechoic source signals. All the signals generated for the scenarios were further filtered with a low pass filter with a cutoff frequency of 10,865 Hz, in order to avoid spatial aliasing effects on the reproduced signals of the planar array, and for conformity.

Five types of binaural signals were generated:

(i) **Reference:** HOA-based reproduction of order 32
(ii) **Low order reference:** First order Ambisonics-based binaural reproduction (*N*=1)
(iii) **BFBR with a sparse beamformer distribution:** BFBR with *D*=4 directions, distributed using a uniform sampling scheme of order *N*_*D*_=1
(iv) **BFBR with the recommended number of beamformers:** BFBR with *D*=12 directions, distributed using a uniform sampling scheme of order *N*_*D*_=2
(v) **BFBR with a dense beamformer distribution:** BFBR with *D*=73 directions, distributed using a nearly uniform sampling scheme of order *N*_*D*_=5

The sampling schemes used for signals (iii), (iv), and (v) were previously detailed in Section 7.5 and were applied to both arrays. It should be noted that no anchor signals were used in this experiment. This is for two reasons. First, no obvious anchor was available for these tests. Second, in order to avoid the case where an extreme anchor would reduce relative differences between conditions in the test. Therefore, signal (ii) was also added in order to better illustrate the results in this section.

Thirteen subjects with no known hearing impairments participated in the listening tests, where 6 of the participants reported to be expert listeners, 5 reported intermediate expertise, and the remaining two were naive listeners. The listening test comprised 8 screens, each including the 5 signals detailed above. In the first 4 screens, which included all four combinations of the two arrays and the two source signals, the subjects were instructed to score the similarity to the reference with respect to spatial attributes. These included a consolidation of externalization, envelopment, horizontal and vertical direction of the source, and spatial disintegration. The following 4 screens were the same, but here, the subjects were instructed to score the similarity to the reference based on timbre perception, or more specifically, the attribute of tone color. All of these attributes were chosen from [51], with each set of 4 screens presented in a random order.

### Results

**Fig. 7:**
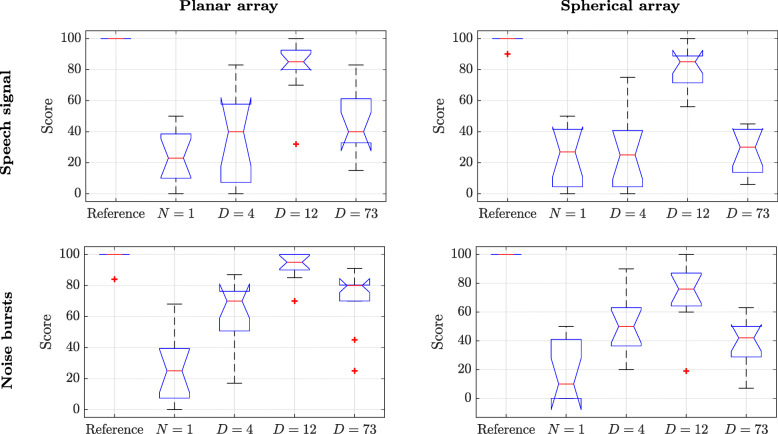
Listening test scores of spatial attributes of the planar and spherical arrays for speech and noise bursts source signals. The signals presented are a high-quality reference HOA-based reproduction of order 32, Ambisonics of order *N*=1, BFBR with *D*=4, BFBR with *D*=12, and BFBR with *D*=73. The boxes show the interquartile range (IQR), for the 25 to 75 percentiles, and the median (red lines). 1.5×*IQR* (whiskers), and 95% confidence levels (notches). Outliers are marked by “+” [52]

**Fig. 8:**
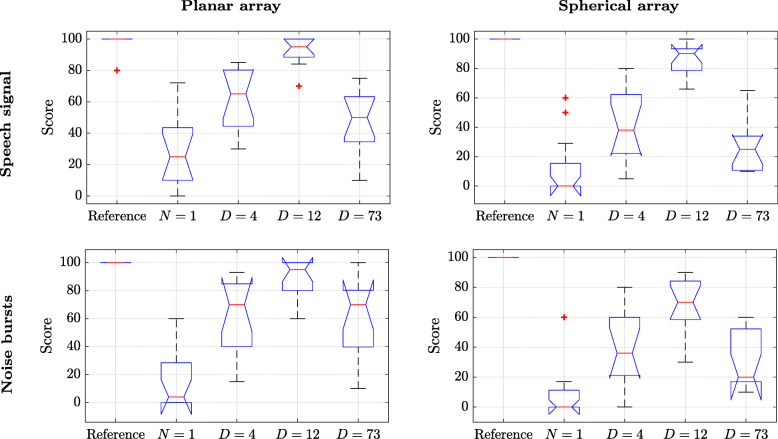
Listening test scores of timbre attributes of the planar and spherical arrays for speech and noise bursts source signals. The signals presented are a high-quality reference HOA-based reproduction of order 32, Ambisonics of order *N*=1, BFBR with *D*=4, BFBR with *D*=12, and BFBR with *D*=73. The boxes show the interquartile range (IQR), for the 25 to 75 percentiles, and the median (red lines). 1.5×*IQR* (whiskers), and 95% confidence levels (notches). Outliers are marked by “+” [52]

The results for the spatial attributes and timbre are presented in Figs. 7 and 8, respectively. Both figures show that the hidden reference signal, i.e., signal (i), was correctly identified by the subjects in all screens, except for a few outliers. Furthermore, the figures show that the *N*=1 signals, i.e., signal (ii), received the lowest scores overall in both tests. Although this signal was not considered to be an anchor in these listening tests, the above result was rather expected since a low SH order is known to limit the perceived spaciousness and to have an effect on the perceived timbre [13]. The BFBR signals show similar trends within each figure. These are discussed below.

A repeated measures analysis of variance (ANOVA) test [53] was performed for both tests. The majority of variables passed the Kolmogorov-Smirnov normality test [53] except for the reference and a few other conditions which failed due to a ceiling effect.

#### Spatial perception

For the screens concerning spatial perception, signal (iv) (BFBR with *D*=12), near the recommended number of directions, is the most similar to the reference in all four screens. The difference in scores between signals (iv) and signals (iii) and (v) (BFBR with *D*=4 and *D*=73, respectively) is significant, as indicated by the box plots. This is true for both arrays and both source signals and demonstrates that the average directivity factor (*D*=9 in this case) is a good measure for the recommended number of beamformers, as suggested in Section 4. The degradation in performance for signal (v) (*D*=73) compared to signal (iv) (*D*=12) is a demonstration of the consequence of choosing too many beamformers, as discussed in Section 5. As discussed, the latter leads to order truncation (see Section 7.4), with impact on spatial perception. As the score for the case of *D*=73 is higher than the score for *N*=1, the order truncation seems to lead to a SH order higher than 1; as is indeed illustrated in Section 7.4, the order of the planar array SH elements of up to order 4 could be identified, where the spherical array is, of course, limited to order 2. This difference between the planar and the spherical arrays may further explain the differences in scores for signal (v) between the two arrays. The scores for signal (iii) with *D*=4 are also significantly lower than for the signals with *D*=12. This is also in line with the theoretical and simulation results presented above. In particular, the use of a too low number of beamformers that only partially cover the entire directional space leads to a noticeable difference from the reference.

A repeated measures ANOVA test, performed for this listening test, resulted in a main effect of the algorithm type independent variable of [ *F*(4,48)=124,*p*<.001]. The anechoic source signal type (speech vs noise bursts) independent variable showed a main effect of [ *F*(1,12)=61.9,*p*<.001], and the array type (planar array vs spherical array) independent variable showed a main effect of [ *F*(1,12)=27.2,*p*<.001]. A sphericity test leads to a correction only for the algorithm type independent variable. Applying the Greenhouse-Geisser correction to the algorithm type independent variable leads to [$F(2.52,30.3)=124, p<.001, \hat {\varepsilon }=.63$]. However, it can be seen from the results that these corrections did not impact significance levels. Figure 7 shows that the trends for the two source signal types are overall similar. The differences between the two array types may be explained by the better performance of signals (iii) and (v) for the planar array compared to that of the spherical array. While the former showed elements up to order 4, the latter has all elements up to order 2 (see Section 7.4).

The interaction between the source signal type and the array type independent variables showed a main effect of [ *F*(1,12)=8.54,*p*=.013], with no necessary correction needed. The interaction between the algorithm type and the source signal type showed a main effect of [ *F*(4,48)=8.02,*p*<.001]. Applying the Greenhouse-Geisser correction to the latter interaction leads to [$F(1.57,18.9)=8.02, p=.005, \hat {\varepsilon }=.39$]. Similarly, the interaction between the algorithm type and the array type showed a main effect of [ *F*(4,48)=6.02,*p*=.001]. Applying the Greenhouse-Geisser correction to the interaction above resulted in a corrected main effect of [$F(2.20,26.4)=6.02, p=.006, \hat {\varepsilon }=.55$]. The interaction between all independent variables showed a main effect of [ *F*(4,48)=1.01,*p*=.41]. Applying the Greenhouse-Geisser correction here leads to a corrected main effect [$F(2.09,25.1)=6.02, p=.38, \hat {\varepsilon }=.52$]. This analysis shows, in particular, that the way in which the binaural reproduction algorithm affects spatial perception may change with array type and signal types, although the trends, as presented in Fig. 7, are similar.

#### Timbre perception

Here, as well, signal (iv) (*D*=12) was scored significantly higher compared to the other signals. Both signal (iii) (*D*=4) and signal (v) (*D*=73) showed a lower score, which demonstrates the theory presented in Sections 4 and 5. Signal (v) (*D*=73) demonstrates the timbre effects due to the use of too many beamformers, while signal (iii) seems to also suffer from timbre distortion due to the use of a too low number of beamformers. The results validate the simulation results presented in Section 7.5.

A repeated measures ANOVA test was also conducted for timbre. Here, this resulted in a main effect for the binaural reproduction algorithm type independent variable of [ *F*(4,48)=98.4,*p*<.001]. The anechoic source signal type independent variable showed a main effect of [ *F*(1,12)=2.90,*p*=.11], and the array type independent variable showed a main effect of [ *F*(1,12)=19.5,*p*=.001]. A sphericity test leads to a correction only for the algorithm type independent variable. Applying the Greenhouse-Geisser correction to the latter lead to [$F(2.77,33.2)=98.4, p<.001, \hat {\varepsilon }=.69$]. The above corrections did not impact significance levels.

The interaction between the source signal type and the array type independent variables showed a non-significant main effect of [ *F*(1,12)=2.68,*p*=.13], with no necessary correction needed. The interaction between the algorithm type and the source signal type showed a main effect of [ *F*(4,48)=7.32,*p*<.001], with a corrected main effect of [$F(2.65,31.8)=7.32, p=.001, \hat {\varepsilon }=.66$]. Similarly, the interaction between the algorithm type and the array type showed a main effect of [ *F*(4,48)=6.24,*p*<.001], with the corrected main effect [$F(3.01,36.1)=6.24, p=.002, \hat {\varepsilon }=.75$]. The interaction between all independent variables showed a main effect of [ *F*(4,48)=2.60,*p*=.047], with the corrected main effect [$F(2.96,35.6)=2.60, p=.068, \hat {\varepsilon }=.74$]. Similarly to the spatial perception listening test, the analysis above also shows that the effects of the binaural reproduction algorithm on timbre may change with array type and with signal types, although the trends, as presented in Fig. 8, are similar here as well.

## Conclusions

In this study, a novel measure for the recommended number of beamformers in BFBR with general arrays was introduced, and the consequences of beamformer direction oversampling with high order HRTFs were investigated theoretically and analyzed numerically. It was demonstrated, both objectively and perceptually, that beamformer direction oversampling leads to spatial degradation and to the attenuation of high frequencies. Furthermore, objective analysis and listening tests showed that by using a number of beamformers near to that recommended herein, the best binaural reproduction is achieved relative to a high-quality reference. Although this study developed an important beamforming measure for BFBR, the conditions under which it is applicable were only evaluated for a single non-spherical array, and were not investigated for a wide range of arrays, beamforming designs, or acoustic scenarios. Furthermore, a comprehensive investigation on the perceptual differences between such arrays, and an investigation of the effects of their DF patterns is also required. Therefore, such studies are proposed for future work. Furthermore, the development of a more comprehensive design methodology for beam pattern design and look direction selection is also proposed for future work.

## Data Availability

The datasets used and analyzed during the current study are available from the corresponding author on reasonable request.

## Abbreviations

ANOVA: Analysis of variance
BFBR: Beamforming-based binaural reproduction
BRIR: Binaural room impulse response
DF: Directivity factor
HOA: Higher order Ambisonics
HRTF: Head-related transfer function
IQR: Interquartile range
MD: Maximum directivity
MUSHRA: MUltiple Stimuli with Hidden Reference and Anchor
MVDR: Minimum variance distortionless response
PWADF: Plane-wave amplitude density function
PWD: Plane-wave decomposition
RIR: Room impulse response
SFT: Spherical Fourier transform
SH: Spherical harmonics
